# Kinematic alignment in total knee arthroplasty restores the native anatomy, with a joint line parallel to the ground on a standing view

**DOI:** 10.1016/j.jor.2025.05.051

**Published:** 2025-05-26

**Authors:** Andrea Pintore, Donato Notarfrancesco, Arnaldo Zara, Francesco Oliva, Filippo Migliorini, Nicola Maffulli

**Affiliations:** a Department of Medicine, Surgery and Dentistry, University of Salerno, Via S. Allende, Baronissi, 84081, Italy; b Casa di Cura Salus, Battipaglia, 84091, Italy; c Department of Human Sciences and Promotion of the Quality of Life, San Raffaele Roma Open University, 00166, Rome, Italy; d Department of Trauma and Reconstructive Surgery, University Hospital of Halle, Martin-Luther University Halle-Wittenberg, 06097, Halle (Saale), Germany; e Department of Orthopaedic and Trauma Surgery, Academic Hospital of Bolzano (SABES-ASDAA), 39100, Bolzano, Italy; f Department of Life Sciences, Health, and Health Professions, Link Campus University, 00165, Rome, Italy; g Department of Trauma and Orthopaedic Surgery, Faculty of Medicine and Psychology, University la Sapienza, 00185, Rome, Italy; h School of Pharmacy and Bioengineering, Keele University Faculty of Medicine, Thornburrow Drive, Stoke on Trent, England, UK; i Queen Mary University of London, Barts and the London School of Medicine and Dentistry, Centre for Sports and Exercise Medicine, Mile End Hospital, 275 Bancroft Road, London, E1 4DG, England, UK

**Keywords:** Knee, Kinematic, Alignment, Total, Arthroplasty, Mechanical

## Abstract

**Background:**

Kinematic alignment (KA) total knee arthroplasty (TKA) restores the native alignment of the knee joint. The present study compared the pre-and post-operative limb alignment of patients undergoing KA-TKA. It evaluated joint line orientation and obliquity on weight-bearing radiographs with the ground as reference.

**Methods:**

56 patients treated with KA-TKA from January to December 2022 were prospectively included. The coronal orientation of the TKA was evaluated on pre- and postoperative anteroposterior long-leg weight-bearing radiographs. For each patient, measurements were made of the hip-knee-ankle (HKA) angle, lateral distal femoral angle (LDFA), medial proximal tibial angle (MPTA), arithmetic HKA (aHKA), and joint line obliquity (JLO).

**Results:**

The mean LDFA of all patients was 84.6° ± 1.7° pre-operatively and 84.5° ± 1.5° post-operatively (P = 0.06). The mean MPTA of all patients was 83.5° ± 1.6° pre-operatively and 85.1° ± 2.1° post-operatively (P = 0.07). The mean HKA of all patients was 172° ± 2° pre-operatively and 175° ± 2° post-operatively (P = 0.47). The difference in LDFA, MPTA, HKA, JLO and aHKA pre-operatively and post-operatively was not statistically significant.

**Conclusion:**

There were no differences in pre- and postoperative radiographic measurements and alignment of the lower limb in patients undergoing KA-TKA, with the joint line orientation parallel to the ground on weight-bearing radiographs in all patients. Longer-term follow-up is necessary to assess the functional outcome and study implant survival of KA-TKA.

## 1 Background

In total knee arthroplasty (TKA), components have traditionally been implanted according to mechanical alignment (MA)^1^ to produce neutral alignment by producing tibial and femoral cuts orthogonal to the mechanical axis.^2^^,^^3^ Despite excellent implant survival,^4^ successful clinical results, and a high patient satisfaction rate,^5^^,^^6^ normal knee function cannot be guaranteed, and residual symptoms may persist,^5^, ^6^, ^7^, ^8^ with 15–20 % of patients being dissatisfied.^9^, ^10^, ^11^, ^12^, ^13^ Several functional knee phenotypes have recently been described, hence the need for a more individualised approach to TKA alignment.^14^ In a recent study, physiological alignment was greater than 3° in varus in nearly one-third of men and nearly 20 % of women.^15^ Coronal alignment is often reported statically as neutral, varus, or valgus and does not consider joint line obliquity (JLO).^16^ The new classification system for the Coronal Plane Alignment of the Knee (CPAK) comprises nine phenotypes that estimate constitutional limb alignment and JLO.^17^ The MA method changes the angle and level of the natural joint line. On the other hand, kinematic alignment (KA) restores the native alignment of the knee joint before the onset of the osteoarthritic process by determining the pre-disease joint line and resecting the femur and tibia parallel to it^18^^,^^19^ as “a personalised joint line reconstruction through anatomic resurfacing, with no ligament releases”.^20^ The proximal tibial joint line is naturally oriented in slight varus, and, during gait, the adduction of the lower limbs brings the joint line parallel to the ground.^21^, ^22^, ^23^ Restoring the patient's native anatomy should also result in a joint line parallel to the ground on weight-bearing radiographs, regardless of whether the physiologic alignment is in the varus or valgus.^24^, ^25^, ^26^, ^27^, ^28^ Favourable outcomes have been reported following KA TKA regarding function, pain relief, feeling of normality, flexion, and implant survival.^29^, ^30^, ^31^, ^32^ The present study compared the pre- and post-operative constitutional limb alignment in patients undergoing KA TKA. The second outcome of interest was joint line orientation and obliquity relative to the ground when standing after KA TKA. The hypothesis was that the joint line orientation would remain parallel to the ground on weight-bearing radiographs in patients undergoing KA TKA.

## 2 Methods

### 2.1 Study design

56 patients admitted to the Department of Orthopaedics of the Casa di Cura Salus of Battipaglia (SA) treated with KA-TKA from January to December 2022 were prospectively included. Before starting this study, all the KA-TKAs were undertaken by the same senior surgeon (D.N.), who was fully trained and experienced in KA-TKA. Patients undergoing a primary TKA in whom conservative management of the underlying Kellgren Lawrence Grade 3 or 4 knee osteoarthritis of the affected knee had been unsuccessful were included. Exclusion criteria were patients undergoing mechanical alignment TKA, revision surgery, severe instability from osteochondral or medial/lateral collateral ligament deficiency, body mass index (BMI) > 35, malunion of fractures of the tibia or femur, and malunion of intra-articular fractures of the distal femur or tibial plateau. All clinical data were obtained from the department's archives. The present study was approved by our local Research Ethics Committee (ID: 0096397, 2021), following the Consolidated Standards of Reporting Trials: the CONSORT statement.^33^ Before surgery, patients gave their written informed consent to the procedure and collection of functional outcomes. They were fully informed of the possible complications of the surgery, accepting also to participate in outcome research. All procedures were performed using a thigh tourniquet. All patients received antibiotic prophylaxis intravenously. The procedure was performed under spinal anesthesia using GMK Sphere Calipered Kinematic Alignment - Medacta International. Plain weight-bearing radiographs were performed post-operatively at discharge.

A standard postoperative rehabilitation regimen was instituted. On the first postoperative day, patients started weight-bearing as able with two elbow crutches and active mobilisation exercises of the operated knee, which they continued during their admission to hospital. Patients were discharged and underwent supervised physiotherapy twice or thrice a week for four weeks post-operatively.

The primary outcome of interest was to compare the pre- and post-operative constitutional limb alignment. The second outcome was to assess joint line orientation and obliquity relative to the ground when weight-bearing.

### 2.2 Radiographic measurements

The results of the plain radiographs and long-leg films before and after surgery were obtained from the hospital's computerised database. The coronal orientation of the TKA was evaluated on pre- and postoperative anteroposterior long-leg weight-bearing radiographs.^21^ For each patient, the hip-knee-ankle (HKA) angle, the lateral distal femoral angle (LDFA), the medial proximal tibial angle (MPTA), the arithmetic HKA (aHKA), and the joint line obliquity (JLO) were measured. The HKA assesses lower limb alignment, defined as the angle formed by the mechanical axes of the femur and the tibia. The LDFA is the lateral angle formed between the mechanical axis of the femur and the joint line of the distal femur. The MPTA was defined as the medial angle formed between the mechanical axis of the tibia and the joint line of the proximal tibia. The aHKA was calculated using the MPTA - LDFA algorithm, which defines the constitutional alignment of the lower limb.^17^ A negative aHKA indicates varus and a positive aHKA indicates valgus constitutional limb alignment. We considered 0° ± 2° boundaries for a neutral aHKA. An aHKA less than −2° defined a varus knee, while an aHKA greater than +2° defined a valgus knee.

The JLO was calculated using the algorithm MPTA + LDFA.^17^ The direction of JLO was classified into three different groups. If these two angles are supplementary (i.e. their sum is 180°), the joint line is approximately neutral. A sum greater than 180° indicates an “apex proximal” joint line; a sum less than 180° indicates an “apex distal” joint line. The parallelism of the joint line relative to the ground was defined in Campo's double-leg weight-bearing stance.

### 2.3 Surgical technique

**Fig. 1:**
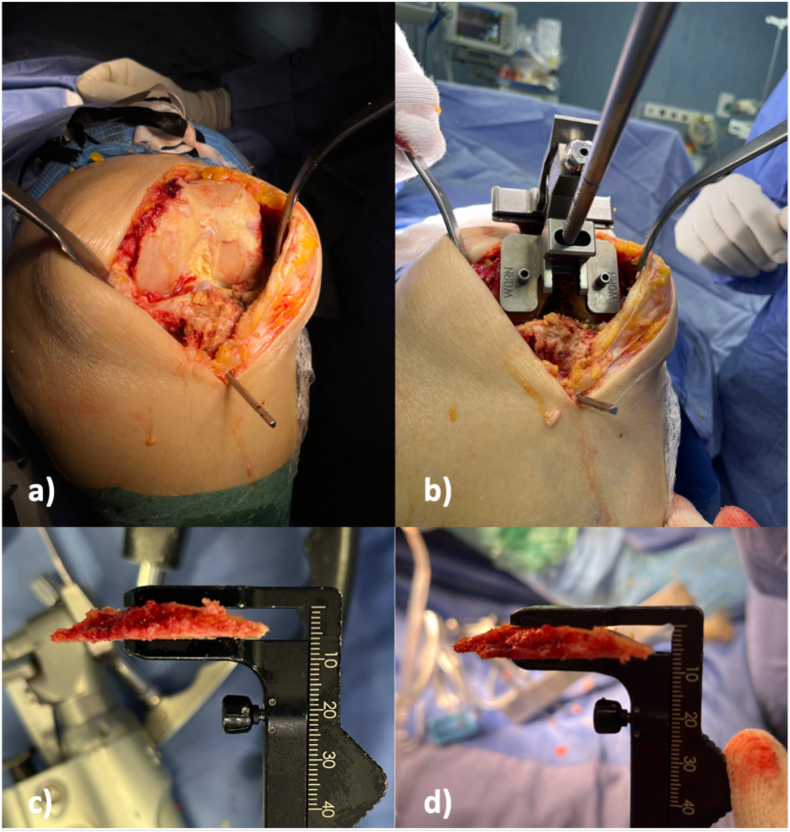
a) Both distal femoral condyles wear. b) Distal referencing guides WORN/WORN. c) Check the thickness of the resected medial and d) lateral distal femoral bone and cartilage resection.

All KA-KAT procedures were performed through a longitudinal midline incision and a medial parapatellar approach, addressing the distal femur first. Calipered kinematic alignment sets the femoral component coincident with the distal articular surface of the native femur. On average, the distal femoral cartilage wear is 2 mm, while varus knees present negligible posterior femoral cartilage wear; the posterior femoral cartilage wear is only 1 mm lateral in valgus knees.^34^ To restore the native distal femoral line, compensations of 2 mm for worn cartilage, when present, on the distal femoral condyles is necessary (Fig. 1). When measuring the cut, the 1 mm thickness of the kerf saw blade should be considered. After inserting the intramedullary rod, the extent of cartilage wear on each distal femoral condyle is determined using a ring curette to remove any partially worn cartilage on the bone. Four distal referencing guides are available: medial cartilage wear, lateral cartilage wear, no cartilage wear on either distal femoral condyle, or both distal femoral condyles (Fig. 1). The distal femoral resection is set at 9 mm, i.e. the thickness of the distal condyles of the femoral component. The dedicated calliper allows to check the thickness of the resection of the medial and lateral distal femur (Fig. 1). The unworn condyles should measure 8.0 ± 0.5 mm versus 6.0 ± 0.5 mm of the worn condyles (Fig. 1). These values are the same as the 9 mm thickness of the distal femoral component condyles after compensating for the 1 mm kerf of the saw blade and 2 mm of worn cartilage, if present.

**Fig. 2:**
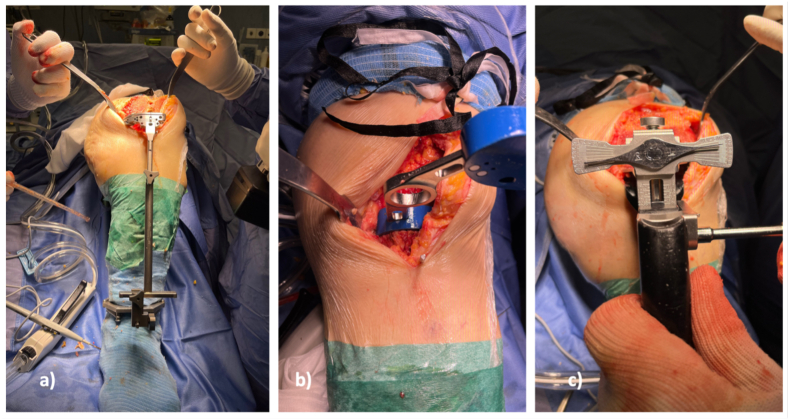
a) The orientation of the tibial resection is parallel to the articular surface of the native tibia. b) and c) Test the flexion and extension gap using the flexion-extension spacers.

Tibial resection is then performed. KA aims to set the tibial component parallel to the flexion-extension axis of the native knee and coincident with the plane of the native tibia. In this way, the varus-valgus and the slope, after compensating for cartilage and bone wear, are restored, maintaining the varus-valgus orientation of the tibial resection parallel to the articular surface of the native tibia (Fig. 2). A 1.27 mm thick saw blade, placed in the slot built into the guide, is used to resect the proximal tibia. The thickness of the resected medial and lateral tibial condyles is measured at the base of the tibial spines, and should be within ±0.5 mm of each other. When one tibial condyle is thinner than the other by 1 mm or more, tightness in that compartment and/or slackness in the other should be expected when varus-valgus laxity is assessed with the knee fully extended. The flexion gap is tested using the flexion-extension spacers.

Subsequently, the cutting block is used to perform the anterior, posterior, and chamfer resections of the femur. The anterior femoral cut is always flush to the anterior cortex to restore the trochlear prominence. The dedicated calliper checks the thickness of the resected medial and lateral posterior femoral resections. The unworn condyles should measure 7.0 ± 0.5 mm, compared to 5.0 ± 0.5 mm of worn condyles. These values equal the 8 mm thickness of the posterior condyles of the femoral component after compensating for the 1 mm kerf of the saw blade and 2 mm of cartilage wear when present.

**Fig. 3:**
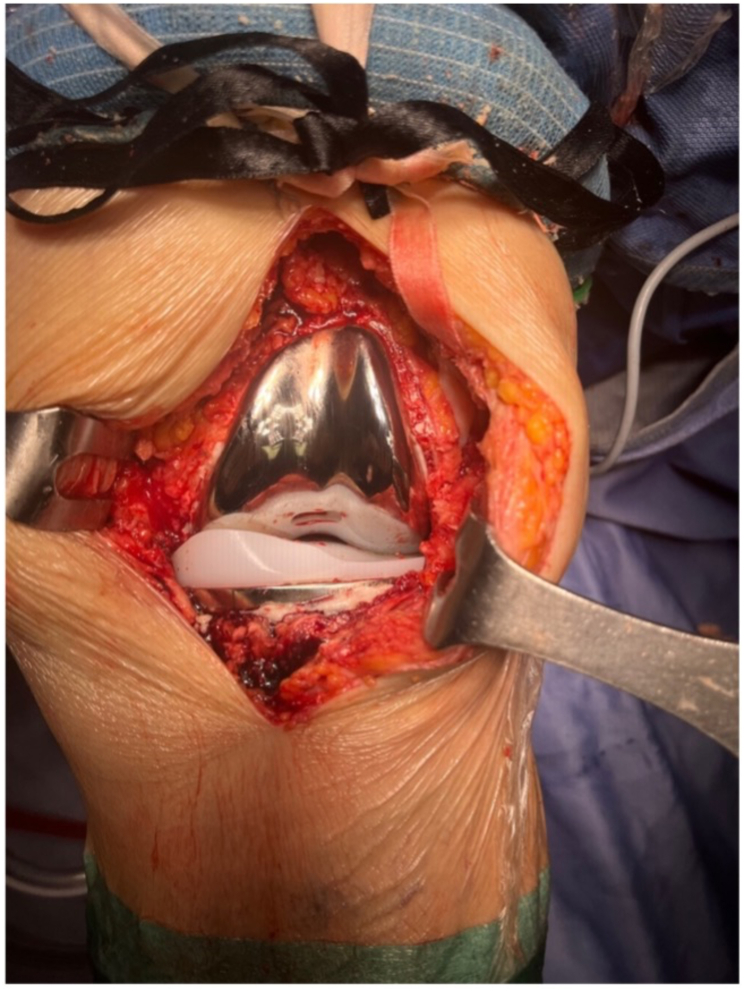
KA-TKA prosthetic components.

The relative tightness between the medial and lateral compartments is checked by internal and external rotation of the spacer. When the knee is flexed to 90°, medial laxity should be lower than lateral laxity, thus matching the native laxity of the medial and lateral compartments of the knee. The spacer should be tight and pivot in the medial compartment and instead be loose in the lateral compartment. This tighter medial/looser lateral fit points to a trapezoid flexion space, as in the native knee. With the knee at 0° of flexion, varus/valgus laxity should be minimal, and the native knee and limb alignments should be re-established. When varus-valgus stress is applied, the medial and lateral gaps should demonstrate equal stability. This shows that both a tight rectangular extension gap and the compartment forces, as in the native knee, have been restored (Fig. 2). The TKA can then be balanced with trial components before refining the preparation of the femur and tibia, resurfacing the patella, and implanting the prosthetic components with bone cement (Fig. 3).

## 3 Results

**Fig. 4:**
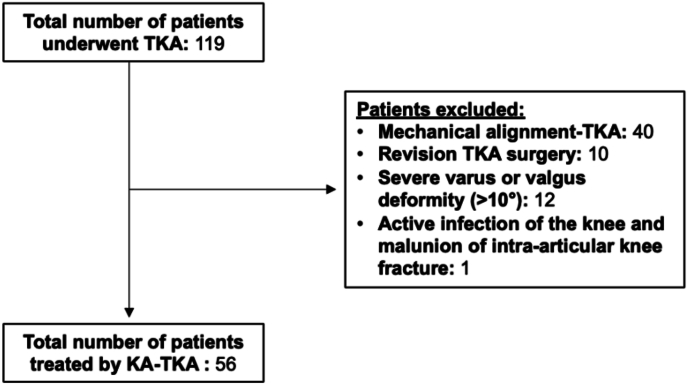
STROBE diagram.

**Fig. 5:**
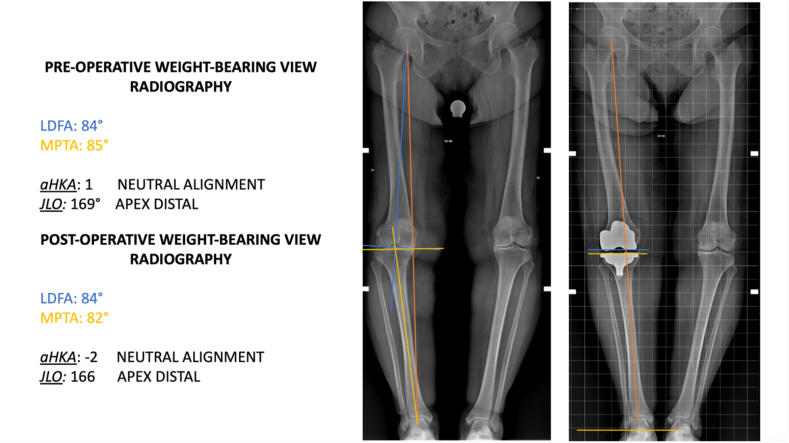
The joint line orientation is parallel to the floor on a weight-bearing radiograph in all patients treated with KA-TKA.

**Fig. 6:**
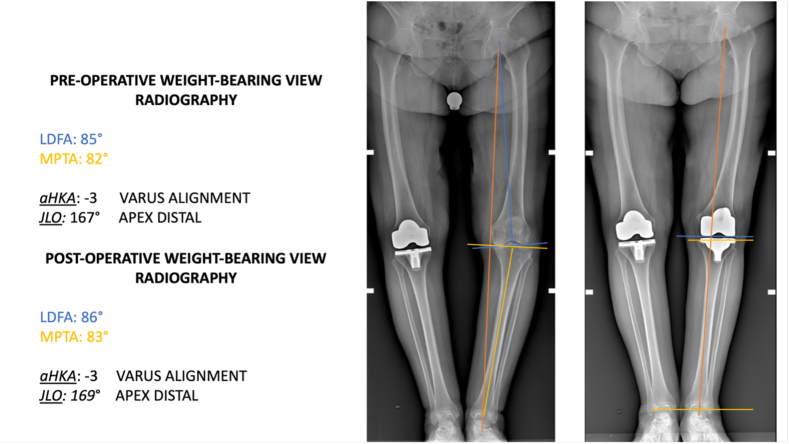
Comparison of the joint line orientation between right knee treated with MA-TKA and left knee treated with KA-TKA.

Between January and December 2022, 119 consecutive patients underwent TKA. Of those, 40 patients were excluded because they underwent mechanical alignment TKA, and ten were excluded because they underwent revision TKA surgery. Twelve patients were excluded because they showed severe instability secondary to advanced osteochondral structure destruction or loss of integrity of the medial or lateral ligament with marked (>10°) varus or valgus deformity. These patients had a deformity >10° with laxity that did not allow ligamentous balancing with a primary prosthetic implant and a medial-pivot insert. One patient was excluded for active infection of the knee and malunion of an intra-articular knee fracture. The remaining 56 patients treated by KA-TKA were included in this study (Fig. 4). Patient demographics are shown in Table 1. The mean LDFA of all patients was 84.6° ± 1.7° pre-operatively and 84.5° ± 1.5° post-operatively (P = 0.06). The mean MPTA of all patients was 83.5° ± 1.6° pre-operatively and 85.1° ± 2.1° post-operatively (P = 0.07). The mean HKA of all patients was 172° ± 2° pre-operatively and 175° ± 2° post-operatively (P = 0.47). The difference in LDFA, MPTA, HKA, JLO and aHKA pre-operatively and post-operatively was not statistically significant. To calculate the alignment of the lower limb expressed by aHKA and obliquity relative to the floor while standing on both legs expressed by JLO, we considered nine knee phenotypes both pre- and post-operatively according to CPAK classification.^17^ The difference in JLO and aHKA between pre-operative and post-operative was not statistically significant (Table 2). The joint line orientation was parallel to the ground on a weight-bearing radiograph in all patients treated with KA-TKA (Fig. 5, Fig. 6).

**Table 1:** Patient demographics.

Number of patients (N)	56
Number of females (%)	46 (82.1)
Mean age (years)	72.6
Mean BMI	25.67
Mean days of admission	4.2
Mean K-L system	3.28

BMI Body mass index; K-L Kellgren and Lawrence.

**Table 2:** Pre- and post-operative radiological measurements of KA-TKA.

	PRE-OPERATIVE			POST-OPERATIVE			P-value^a^
**LDFA**^b^	84.6 ± 1.7			84.5 ± 1.5			0.06
**MPTA**^b^	83.5 ± 1.6			85.1 ± 2.1			0.07
**HKA2**^b^	172 ± 2			175 ± 2			0.47

	**aHKA VARUS (%)**	**aHKA NEUTRAL (%)**	**aHKA VALGUS (%)**	**aHKA VARUS (%)**	**aHKA NEUTRAL (%)**	**aHKA VALGUS (%)**	

**JLO APEX DISTAL**^c^**(%)**	23 (50)	17 (37)	6 (13)	20 (42.5)	21 (44.7)	5 (12.8)	
**JLO NEUTRAL**^c^**(%)**	5 (50)	3 (30)	2 (20)	5 (50)	4 (40)	1 (10)	
**JLO APEX PROXIMAL**^c^**(%)**	0 (0)	0 (0)	0 (0)	0 (0)	0 (0)	0 (0)	

LDFA lateral distal femoral angle; MPTA medial proximal tibial angle; HKA hip-knee-ankle angle; JLO joint line obliquity; aHKA Arithmetic hip-knee angle.

a p value is calculated between pre-operative and post-operative results.

b Data was expressed by mean ± standard deviation.

c Data was expressed by number of patients (% of patients).

## 4 Discussion

In 2018, Howell et al. showed an implant survival rate of 98.4 % at a 10-year follow-up of 222 kinematically aligned TKA.^35^ In a randomised controlled trial, KA-TKA led to significantly greater improvement in quantitative variables of knee balance than MA-TKA.^36^ A prospective observational study noted that patients with constitutional varus knees had a significantly higher risk than non-varus knees for OA to advance.^37^ KA aims to restore the pre-constitutional varus in the knee before it develops osteoarthritic changes, decreases soft tissue adjustment, and restore normal knee kinematics.^29^^,^^38^ KA-TKA aims to restore the native tibial-femoral articular surfaces, restore the native limb and knee alignments, and restore the native laxity of the knee.^39^ This can be performed through anatomical femoral resurfacing, a tibial cut orientation in slight varus, and no ligament releases. The KA technique also aims to restore patient-specific patella kinematics. However, the femoral implant used in KA-TKA has the limitation of restoring the femoral morphology without considering the anatomical differences of the medial and lateral condyles. This may influence the patellofemoral kinematics. The current evidence shows no catastrophic patellofemoral failure. In the mid-term, patellar loosening and patellar instability in KA-TKA were low (0.9 % and 0.4 %–1.4 %, respectively),^40^, ^41^, ^42^, ^43^, ^44^ and anterior knee pain was five times less likely^32^ than in MA-TKA. The medial-pivot insert can reproduce the normal biomechanics of the knee, with a slight lateral laxity at 90° of flexion and no laxity at 0°of flexion.^45^^,^^46^ Commonly, an arthritic knee deforms and accentuates the original mechanical axis of the lower limb. This results in a change of mechanical hip-knee-ankle angle but no change of LDFA and MPTA, and consequently of aHKA.^16^^,^^47^ For this reason, our results compared post-operative limb alignment with the native constitutional alignment of each patient, not influenced by arthritic degenerative deformity.

The difference in LDFA, MPTA and HKA before and after the index procedure was not statistically significant. The pre-operative and post-operative differences between JLO and aHKA were not statistically significant. The knee phenotypes showed no differences pre- and post-operatively according to the CPAK classification.

Recently, a meta-analysis of randomised controlled clinical trials, which included 1103 patients followed up for 6–24 months, compared KA and MA in TKA.^48^ The KA-TKA group fared better than the MA-TKA group regarding clinical outcomes and knee ROM. In contrast, complications, HKA, LDFA, and MPTA angle in the KA-TKA group were not significantly different from those in the MA-TKA group. Regarding perioperative results, a meta-analysis showed that patients who received KA-TKA technique exhibited a longer walking distance before discharge than with MA-TKA.^49^ This might also explain why patients with KA-TKA are generally more satisfied than those with MA-TKA.^44^ However, the follow-up of patients treated by KA-TKA is still relatively short, and longer-term investigations with more RCTs and multicentric studies are necessary to evaluate implant survival with KA-TKA.

KA-TKA may not be indicated in all patients requiring TKA.^50^ Extra-articular deformities producing a pathological JLO and pathological laxity of the collateral ligaments can be considered contraindications to KA-TKA.^51^ In contrast to varus type OA, valgus deformities appear more critical for KA.^39^ Indeed, the indications for KA in severe valgus knees are uncertain, especially when excessive medial collateral ligament stretching, extreme knock knees, or lateral patella subluxation are present.^52^ In patients with atypical or extreme knee anatomy, the restricted-KA protocol is an alternative to classical KA-TKA,^53^ aiming to reproduce the constitutional knee anatomy within a safe range.^54^ The safe range should retain HKA between −3° and 3°, and MPTA and LDFA between 87° and 93°. For these patients, a computer- or robotic-assisted TKA and MCL soft-tissue release are more likely to produce acceptable results.^55^^,^^56^ Furthermore, the asymmetric polyethylene thickness as available on some implants may increase the safe range in restricted-KA.

This study has some limitations. For example, we studied our patients in a prospective fashion and did not include a control group, such as a comparison with MA-TKA. To confirm the alignment of the knee, the obliquity of the joint line could also be measured with the contralateral side. Another important limitation was that our results considered only radiographic measurements. Then, the ankle distance is not always controlled in 3-foot films, and this could have an impact on the joint line angle relative to the ground. However, the recruitment process was scrupulous, data collection proceeded in a rigorous scientific fashion, we used reliable, validated radiographic measurements, and the results obtained carried clinical relevance. On the other hand, to our knowledge, no studies have previously compared pre-operative and post-operative radiographic results for KA-TKA. Longer-term follow-up is necessary to investigate the functional outcome and implant survival of KA-TKA better.

## 5 Conclusion

Our study showed no difference in pre-and postoperative radiographic measurements and lower limb alignment in patients undergoing KA-TKA, who exhibited a joint line orientation parallel to the ground on weight-bearing radiographs. Longer-term follow-up studies are necessary to confirm the functional outcomes and implant survival of KA-TKA.

## Consent to participate

The present study was conducted in accordance to principles in the Declaration of Helsinki. All subjects and/or their legal guardian(s) gave their written informed consent.

## Registration and protocol

The present review was not registered.

## Author contribution statement

AP: writing; DN: writing; AZ: writing; FO: revision; FM: revision; NM: supervision. All authors agreed to the final version to be submitted for publication, and are accountable for the whole work.

## Ethical approval

The present study was approved by the South Campania Ethics Committee (ID: 0096397, December 20, 2021).

## Availability of data and materials

The datasets generated during and/or analysed during the current study are available at reasonable request to Dr Andrea Pintore (apintore@unisa.it).

## Funding

No financial support was received for the research, authorship, and/or publication of this manuscript.

## Competing interests

The authors declare no competing interests for this article.

## List of Abbreviations

• TKA: Total Knee Arthroplasty
• KA: Kinematic Alignment
• MA: Mechanical Alignment
• HKA: Hip-Knee-Ankle (angle)
• LDFA: Lateral Distal Femoral Angle
• MPTA: Medial Proximal Tibial Angle
• aHKA: Arithmetic Hip-Knee-Ankle (angle)
• JLO: Joint Line Obliquity
• CPAK: Coronal Plane Alignment of the Knee
• RCT: Randomised Controlled Trial
• OA: Osteoarthritis
• BMI: Body Mass Index
